# Presence of the knockdown resistance mutation, *Vgsc-1014F* in *Anopheles gambiae* and *An. arabiensis* in western Kenya

**DOI:** 10.1186/s13071-015-1223-5

**Published:** 2015-12-01

**Authors:** Eric Ochomo, Krishanthi Subramaniam, Brigid Kemei, Emily Rippon, Nabie M. Bayoh, Luna Kamau, Francis Atieli, John M. Vulule, Collins Ouma, John Gimnig, Martin J. Donnelly, Charles Mbogo

**Affiliations:** School of Public Health and Community Development, Maseno University, Maseno, Kenya; Centre for Global Health Research, Kenya Medical Research Institute, P. O. Box 1578, Kisumu, 40100 Kenya; Department of Vector Biology, Liverpool School of Tropical Medicine, Liverpool, UK; Centre for Biotechnology and Research Development, Kenya Medical Research Institute, Nairobi, Kenya; Health Challenges and Systems, African Population and Health Research Centre, Nairobi, Kenya; Centers of Disease Control and Prevention, Atlanta, USA; Malaria Programme, Wellcome Trust Sanger Institute, Cambridge, UK; Kenya Medical Research Institute, Centre for Geographic Medicine Research-Coast, Kilifi, Kenya; Malaria Public Health Department, KEMRI-Wellcome Trust Research Program, Nairobi, Kenya

**Keywords:** *Kdr*, Insecticide resistance, Pyrethroids, *Anopheles gambiae*

## Abstract

**Introduction:**

The voltage gated sodium channel mutation *Vgsc-1014S* (*kdr*-east) was first reported in Kenya in 2000 and has since been observed to occur at high frequencies in the local *Anopheles gambiae* s.s. population. The mutation *Vgsc-1014F* has never been reported from *An. gambiae* Complex complex mosquitoes in Kenya.

**Findings:**

Molecularly confirmed *An. gambiae s.s.* (hereafter *An. gambiae*) and *An. arabiensis* collected from 4 different parts of western Kenya were genotyped for *kdr* from 2011 to 2013. *Vgsc-1014F* was observed to have emerged, apparently, simultaneously in both *An. gambiae* and *An. arabiensis* in 2012. A portion of the samples were submitted for sequencing in order to confirm the *Vgsc-1014F* genotyping results. The resulting sequence data were deposited in GenBank (Accession numbers: KR867642-KR867651, KT758295-KT758303). A single *Vgsc*-*1014F* haplotype was observed suggesting, a common origin in both species.

**Conclusion:**

This is the first report of *Vgsc-1014F* in Kenya. Based on our samples, the mutation is present in low frequencies in both *An. gambiae* and *An. arabiensis*. It is important that we start monitoring relative frequencies of the two *kdr* genes so that we can determine their relative importance in an area of high insecticide treated net ownership.

## Introduction

The two most widely applied vector control tools, insecticide treated nets (ITNs) and indoor residual spraying (IRS) have contributed greatly to the decline in global malaria rates [1, 2]. Pyrethroids are the most commonly used insecticides in control programs due to their low human toxicity and high efficacy against vectors [3, 4]. Previously, DDT, an organochlorine, was the most widely used insecticide for vector control with its use spread out over multiple countries for malaria control [5, 6]. The widespread use of these insecticides has likely contributed to the selection of resistance across sub-Saharan Africa [7] (http://www.irmapper.com/).

Increased resistance to pyrethroids is particularly troubling since this is the only class of insecticides approved by WHO for use on ITNs [3]. If ITNs are rendered ineffective, a surge in malaria transmission could follow [8]. Resistance to pyrethroids has been reported from multiple sites in western Kenya [9, 10] with both target site and metabolic resistance mechanisms implicated [9–15]. DDT and pyrethroids function by binding to the voltage gated sodium channels (*Vgsc*) on the mosquito’s neurons delaying the closing of the sodium channel; prolonging the action potential and causing repetitive neuron firing, ultimately resulting in paralysis and death [8, 16].

In *Anopheles gambiae s.l.,* knock down resistance (*kdr*) is commonly caused by mutations in the *Vgsc-* either from leucine (TTA) to phenylalanine (TTT) or leucine to serine (TCA) [11, 17] at codon 1014. *Vgsc-1014S* (formerly *kdr*-east) was first reported in Kenya in 2000 and has been observed to occur at high frequencies in the local *An. gambiae* populations [10, 11]. Thus far, there has been no report of the existence of *Vgsc-1014F* (formerly *kdr*-west) in Kenya but has been reported in Uganda and Tanzania in the recent past [18, 19]. Our work demonstrates the emergence of *Vgsc-1014F* in western Kenya in two principal malaria vectors, *An. gambiae* and *An. arabiensis*.

## Findings

### Material and methods

This study was conducted in four malaria endemic districts in western Kenya with two distinct Vector control interventions: Rachuonyo and Nyando where IRS (Deltamethrin in 2011 and lambdacyhalothrin in 2012) was combined with ITNs (treated with permethrin or deltamethrin); and in Bondo and Teso where only ITNs are deployed [9]. Mosquito collections were performed annually between June and September in 2011, 2012 and 2013. Mosquito sampling, rearing and bioassays of emergent adults were conducted as described in Ochomo et al. [9].

### Species identification & Vgsc genotyping

DNA was extracted from whole specimens and a PCR assay [20] was used to distinguish between *An. gambiae* and *An. arabiensis*. DNA samples were genotyped to identify the *kdr* genotype at amino acid position 1014 of the *Vgsc* using a modification of the protocol by Bass et al., [21] as described in Mathias et al., [10].

### Exon sequencing of *Vgsc*

Previous studies in western Kenya have only reported the presence of *Vgsc-1014S* mutation. Therefore, in order to confirm the presence of *Vgsc-1014F* and to determine if was a *de novo* origin, a subsample of the *Vgsc-1014F* carriers were Sanger sequenced. Prior to sequencing, conventional PCR was used to amplify the exon 20 [22] which contains the 1014 locus^].^ Samples were sequenced at Centre for Genomic Research, University of Liverpool, UK and resulting sequences aligned using CodonCode aligner (http://www.codoncode.com/aligner/).

### Analysis for the origin of *Vgsc-1014F* Mutation

Gene sequences obtained from the sequencing exons 20 and 27 were aligned using Codon Code aligner (http://www.codoncode.com/) and the contigs transferred to DnaSP (http://www.ub.edu/dnasp/) as FASTA files. The files were concatenated and then run using the PHASE algorithm in DnaSP [23]. The phased files were exported as a Phylip file to TCS, a statistical parsimony software for phylogenetic network estimation (http://darwin.uvigo.es/software/tcs.html).

## Results

### Frequency of *Vgsc-1014S* and *Vgsc-1014F* in the study sites from 2011 to 2013

We observed low frequencies of *Vgsc-1014S* in *An. arabiensis,*even though we had high frequencies of the same allele in *An. gambiae*, they were much lower than has been reported previously [10]. We saw a simultaneous appearance of *Vgsc-1014F* inboth *An. gambiae* and *An. arabiensis* in 2012 in all four study sites (Table 1) and thereafter compared the mean frequencies of the genes among the three years (Table 2). Of these, 19 samples (12 *An. gambiae* and 7 *An. arabiensis*) were sequenced. 3 *An. gambiae* and 3 *An. arabiensis* were confirmed to be homozygous for *Vgsc-1014F* with one *An. arabiensis* heterozygote detected. The sequences were deposited in GenBank (Accession numbers: KR867642- KR867651, KT758295-KT758303).

**Table 1:** Frequency of *Vgsc-1014F* and *Vgsc-1014S* mutations in *An. gambiae* and *An. arabiensis* populations of western Kenya from 2011 to 2013

		*An. arabiensis*			*An. gambiae*		
District	Year	*N*	*Vgsc_1014S*	*Vgsc_1014F*	*N*	*Vgsc_1014S*	*Vgsc_1014F*
Bondo	2011	105	0.052	0	0		
	2012	129	0.031	0.047	2	0	0
	2013	236	0.008	0.125	2	0	0
Nyando	2011	284	0.016	0	0		
	2012	82	0.012	0.024	1	0	0
	2013	173	0.055	0.023	5	0	0
Rachuonyo	2011	20	0.05	0	0		
	2012	53	0	0.047	1	0	0
	2013	136	0.018	0.011	5	0	0
Teso	2011	7	0	0	211	0.94	0
	2012	4	0	0	189	0.68	0.054
	2013	60	0.019	0	317	0.85	0.025

**Table 2:** Comparison of mean frequencies of *Vgsc-1014F* and *Vgsc-1014S* mutations in *An. arabiensis* using ANOVA and Tukey’s test. A similar analysis could not be done for *An. gambiae* as only one site (Teso) had sufficient numbers of *An. gambiae*

	Vgsc-1014F				Vgsc-1014S			
Year	Difference	Lower limit	Upper limit	Adjusted *P*-value	Difference	Lower limit	Upper limit	Adjusted *P*-value
2011–2012	0.043	−0.019	0.105	0.186	−0.084	−0.877	0.709	0.953
2011–2013	0.046	−0.016	0.108	0.152	−0.032	−0.824	0.761	0.993
2012–2013	0.003	−0.059	0.065	0.99	0.052	0.741	0.845	0.982

**Fig. 1:**
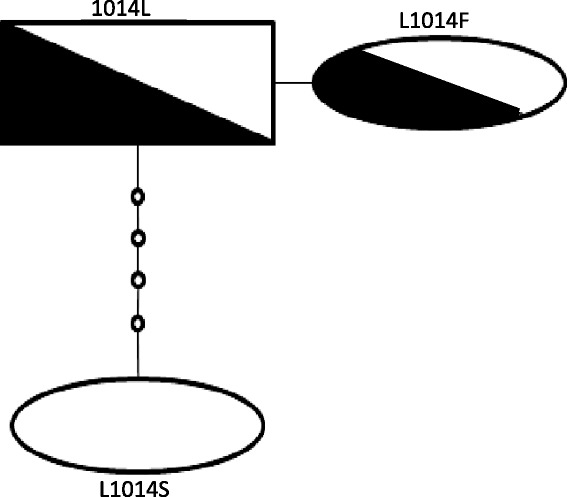
A TCS plot of the three haplotypes present in the populations assayed. White colour represents *An. gambiae* while black colour represents *An. arabiensis*

Only a single 1014F haplotype was observed (Fig. 1), suggesting a common origin in the species and subsequent interspecific transfer. However it should be noted that our ability to resolve different haplotypes was constrained by the low levels of diversity at this locus and our small amplicon length (478bp).

## Discussion

This is the first report of *Vgsc-1014F* in Kenya, which appears to have emerged in both *An. gambiae* and *An. arabiensis* around 2012 and is confirmed via DNA sequencing in multiple samples. The gene has previously been observed in Uganda [18], then much later in Tanzania [19] and now in Kenya. We have developed this report to alert researchers and programmatic staff to the presence of *Vgsc-1014F* mutation in these two important *Anopheles* vectors so that they can modify their resistance marker screening procedures. It is important therefore that we start monitoring allele and genotype frequencies so that we can assess their impact in an area of high bednet ownership.
